# Editorial: Cognition and mobility with aging or neurological conditions: Assessment and interventions strategies

**DOI:** 10.3389/fneur.2022.1089584

**Published:** 2022-12-02

**Authors:** Maud Ranchet, Laurence Paire-Ficout, Hannes Devos

**Affiliations:** ^1^Health, Safety and Transport Department - Laboratory Ergonomics and Cognitive Sciences Applied to Transport (TS2-LESCOT), University of Gustave Eiffel, The French Institute of Science and Technology for Transport, Development and Networks (IFSTTAR), University of Lyon, Lyon, France; ^2^Department of Physical Therapy and Rehabilitation Science, School of Health Professions, The University of Kansas Medical Center, Kansas City, KS, United States

**Keywords:** cognition, mobility, aging, neurological conditions, interventions

## Introduction

Aging and neurological conditions may impact everyday mobility activities. Age-related cognitive decline may be associated with difficulties with mobility is essential to avoid social isolation and negative consequences on quality of life. Different types of interventions (cognitive training, aerobic exercise training, and educational programs) may maintain or improve cognition and mobility in older individuals or individuals with neurological conditions.

Therefore, we host this special Research Topic for Frontiers in Neurology and Frontiers in Neurorehabilitation that focuses on cognition and mobility with aging and neurological conditions. The aim of this Research Topic is to share and discuss recent advances to better assess and potentially improve cognition and/or mobility in older adults or adults with neurological disorders.

This Research Topic includes 15 manuscripts: 11 original research articles, two systematic reviews, one perspective paper, and one study protocol. The papers cover three main domains: (1) cognition; (2) mobility; and (3) interactions between cognition and mobility.

## Cognition

Establishing reliable tests to measure different cognitive functions following a stroke is essential for clinicians' practices and for improving scientific knowledge. The Oxford Cognitive Screen (OCS) is a screening tool that provides a “snapshot” of a patient's cognitive profile. To foster the usability of OCS for both clinicians and researchers, Iosa et al. proposed a new visual snapshot that expresses OCS sub-tests as a function of the six cognitive domains: language and arithmetic, memory, visuomotor control, orientation, spatial exploration, and executive functions.

Functional cognitive assessments may be complementary to neuropsychological tests to assess cognition in stroke patients. As an example, Jaywant et al. assessed a 10-item, short-form Weekly Calendar Planning Activity (WCPA-10) as a cognitive instrumental activity of daily living, in patients who had a stroke and were undergoing acute rehabilitation. This tool involves entering a list of simulated, fictional appointments into a weekly schedule while keeping track of, and adhering to multiple task rules and ignoring built-in obstacles and distractions. The results showed that WCPA-10 captures functional performance deficits in stroke patients. This study highlights the need to use performance based, functional cognitive assessments, even for those who perform well on cognitive screening tools.

In the study by Park et al., machine learning models were used to evaluate whether a comprehensive visual rating scale, based on magnetic resonance imaging, can predict progression to dementia. The authors found that tree-based machine learning algorithms outperformed logistic regression in predicting conversion from mild cognitive impairment to dementia, based on features of the comprehensive visual rating scale and clinical data.

## Mobility

Four papers evaluated the effects of aging and neurological conditions on the spectrum of mobility, including gait, driving, and autonomous transportation. Two studies proposed new interventions to promote mobility in patients with neurological conditions.

Jiang et al. presented the Composite Activity-related Risk of Falls Scale (CARFS). This scale is designed to measure the risk of falls in relation to the activity-specific fear of falling and physical behavior. The paper attests to the reliability and validity of the CARFS in older people with various medical conditions and persons who had a stroke or spinal cord injury.

In a perspective paper, Dale et al. described the feasibility of standardized objective balance assessments in individuals with progressive supranuclear palsy (PSP). The authors encourage safe practices to facilitate more objective balance testing in individuals with PSP.

In a systematic review and meta-analysis, Karpodini et al. evaluated the pooled evidence from 18 randomized controlled trials, comparing the effect of rhythmic auditory stimulation to a control intervention on gait in individuals with Parkinson's disease. The results showed a beneficial effect of rhythmic auditory stimulation on gait, mobility, and quality of life.

Classen et al. investigated the readiness of older adults to accept autonomous shuttles as a mode of transportation. A total of 104 older drivers completed an Automated Vehicle User Perception Survey before and after journeying in an automated shuttle. Technology readiness and barriers to autonomous vehicles were the main predictors of the intention to use the automated shuttle.

Older drivers with cognitive disorders may benefit from interventions to improve their on-road driving safety. As an example, George et al. evaluated the effect of a group-based support and education program (the CarFreeMe TI program) on community mobility (e.g., the use of public transportation) for 20 individuals with traumatic brain or spinal cord injuries who cannot fully return to driving. Although this program did not increase the number of outings away from home, the individuals who received the intervention were more likely to use public transport and transport services, and had an improved quality of life when compared with individuals who received information related to transport options (control group).

In a study protocol, Delphin-Combe et al. proposed an innovative therapeutic educational program (the ACCOMPAGNE program) for patient/natural caregiver dyads who wish to implement self-regulation strategies in driving activity and to improve self-awareness of a patient's driving ability. Awareness has been suggested as a key motivator in compensatory behavior regarding modifications to driving performance.

## Interactions between cognition and mobility

Four papers investigated the associations between cognitive performance and mobility measures. Locomotor adaptation, i.e., the ability to adjust stepping movements to changing environmental demands, is essential to walk safely in constantly changing environments.

Pottorf et al. revealed that older individuals with mild cognitive impairment and Alzheimer's Disease showed a reduced magnitude of locomotor adaptation, particularly during the early adaptation phase of split-belt walking. Interestingly, the authors found associations between reduced locomotor adaptation and cognitive impairments, suggesting that individuals who have cognitive impairments may also demonstrate impairments in locomotor adaptation.

Another study examined the associations between turning mobility and cognitive functions in patients with chronic post-stroke symptoms (Kuan et al.). The authors found that turning mobility was significantly associated with global cognitive function and distinct cognitive domains, such as visuospatial ability and language. The authors concluded that stroke patients, with poorer cognition, impairments in language, or visuospatial ability, may be more prone to instability when performing walking turns or turning to the paretic side.

Lee et al. evaluated changes in dual-task performance after robotic upper extremity rehabilitation in individuals who had a hemiplegic stroke. After 4 weeks of robotic rehabilitation, participants improved more in single motor tasks than in single cognitive tasks. The benefits of robotic rehabilitation on motor outcomes were even more evident in the dual-task conditions. After a mild traumatic brain injury (mTBI), patients may report imbalance during walking, with head movements caused by injury to the vestibular system. D'Silva et al. showed that after a mTBI, people may exhibit a slower usual gait speed compared with age-matched controls. In particular, with head turns and an added cognitive task, their gait speed decreased and continued to be significantly slower than the healthy controls. The authors highlight the important implications for people with mTBI as they return to work, leisure, and community activities.

## Conclusion

Assessing cognitive functions, especially executive functions, in a real-life context in different populations of people with neurological conditions should be further explored, as they play a major role in everyday mobility activities. Furthermore, there is now a large body of evidence indicating that interventions (e.g., educational intervention or training programs) may help older drivers and those with cognitive impairments to maintain their mobility and quality of life. Finally, with health and environmental issues, changes in daily mobility are observed, especially in urban areas. A better understanding of the associations between cognitive functions and different modes of mobilities (walking, cycling, or riding a personal mobility device) could help to design new interventions to promote active mobility in older adults and those with neurological impairments.

## Author contributions

All authors listed have made a substantial, direct, and intellectual contribution to the work and approved it for publication.

## Conflict of interest

The authors declare that the research was conducted in the absence of any commercial or financial relationships that could be construed as a potential conflict of interest.

## Publisher's note

All claims expressed in this article are solely those of the authors and do not necessarily represent those of their affiliated organizations, or those of the publisher, the editors and the reviewers. Any product that may be evaluated in this article, or claim that may be made by its manufacturer, is not guaranteed or endorsed by the publisher.

